# An Application of Outer Membrane Protein P6-Specific Enzyme-Linked Immunosorbent Assay for Detection of *Haemophilus influenzae* in Middle Ear Fluids and Nasopharyngeal Secretions

**DOI:** 10.1371/journal.pone.0071774

**Published:** 2013-08-28

**Authors:** Muneki Hotomi, Akihisa Togawa, Masamitsu Kono, Gen Sugita, Rinya Sugita, Yutaka Fujimaki, Yosuke Kamide, Akihiro Uchizono, Keiko Kanesada, Shoichi Sawada, Naohiro Okitsu, Hisayo Masuda, Hideaki Tanaka, Yumi Tanaka, Noboru Yamanaka

**Affiliations:** 1 Department of Otolaryngology-Head and Neck Surgery, Wakayama Medical University, Wakayama, Japan; 2 Sugita ENT clinic, Chiba, Japan; 3 Fujimaki ENT clinic, Chiba, Japan; 4 Kamide ENT clinic, Shizuoka, Japan; 5 Sendai ENT clinic, Kagoshima, Japan; 6 Nonohana ENT clinic, Yamaguchi, Japan; 7 Sawada ENT clinic, Kochi, Japan; 8 Department of Otolaryngology, Tohoku Rosai Hospital, Miyagi, Japan; 9 Otsuka Pharmaceutical Co., Ltd, Tokyo, Japan; Charité, Campus Benjamin Franklin, Germany

## Abstract

An enzyme-linked immunosorbent assay specific to outer membrane protein P6 (P6-ELISA) was applied for detecting *Haemophilus influenzae* in middle ear fluids (MEFs) from acute otitis media (AOM) patients and in nasopharyngeal secretions (NPSs) from acute rhinosinusitis patients. P6-ELISA had a sensitivity of 83.3% for MEFs and 71.5% for NPSs and a specificity of 85.6% for MEFs and 92.5% for NPSs, respectively. Real-time PCR exhibited significant differences in the number of *ompP1* gene copies among samples determined by P6-ELISA to be positive and negative for *H. influenzae*. However, because the P6-ELISA test has the reactivity in *Haemophilus* species include two commensals *H. haemolyticus* and *H. parainfluenzae*, it is thus a weak method in order to detect only NTHi correctly. Consequently, diagnosis using the P6-ELISA should be based on an overall evaluation, including the results of other related examinations and clinical symptoms to prevent misleading conclusions in clinical setting.

## Introduction

*Haemophilus influenzae* is one of the normal inhabitants of the human nasopharynx and is responsible for acute otitis media (AOM) as well as acute rhinosinusitis [1]. Over 90% of isolates responsible for these infectious diseases are nontypeable strains with deficient capsular polysaccharide, while most cases of severe invasive disease such as meningitis and sepsis were attributable to type b encapsulated strains [2].

Early diagnosis is important for appropriate antimicrobial treatment of infectious diseases, with bacterial culture continuing to be the gold standard of diagnostic methods. However, it is common for bacterial cultures to show false negatives. Accurate diagnosis of infections caused by *H. influenzae* is often difficult because the quality of samples appropriate for culture is frequently reduced by the presence of inflammatory products and/or antibiotics. *H. influenzae* is highly auxotrophic and facultatively anaerobic and requires carbon dioxide and X and V factors for its growth [2], [3]. These bacteria frequently perish immediately from drying during the process of sample collection and culture. Treatment of patients with antimicrobial drugs prior to specimen collection and anti-inflammatory components included in specimens both strongly reduce the growth of the pathogens collected for conventional bacterial culture techniques. Furthermore, a recent study shows that conventional microbiology does not readily distinguish nontypeable *H. influenzae* (NTHi) from its close nonpatogenic relatives such as *H. haemolyticus* and *H. parainfluenzae*. Adding to the difficulties of accurate diagnosis is the recent increase of ß-lactamase non-producing ampicillin-resistant (BLNAR) strains of *H. influenzae*, which require rapid and accurate identification if appropriate antimicrobial treatments are to prevent persistent or recurrent infections [4]–[6].

A variety of tests have been applied to the detection of *H. influenzae*: Latex agglutination, counterimmunoelectrophoresis, coagglutination, and enzyme-linked immunosorbent assay (ELISA) for capsular polysaccharide have been applied to diagnose type b *H. influenzae* [7]–[9]. However, in routine clinical practice, these immunologic diagnostic tools based on polysaccharides antigens are not appropriate for the detection of nonencapsulated nontypeable strains.

The outer membrane protein (OMP) P6 of *H. influenzae* is a peptideglycan-associated lipoprotein at 16600 dalton [10]–[12]. Its encoding gene exhibits a high degree of sequence conservation among strains, and its antigenicity is stable [13]. Consequently, P6 is considered an ideal diagnostic target as well as an attractive vaccine candidate for *H. influenzae*. Our successful application of PCR for detecting the P6 gene in middle ear fluids (MEFs) has been described elsewhere [14]. However, this technique has some limitations in routine clinical practice [14], [15]. In the present study, we evaluated P6-specific ELISA (P6-ELISA) for the direct detection of *H. influenzae* in MEFs and nasopharyngeal secretions (NPSs).

## Material and Methods

### Study populations

This study was conducted in Japan between December 2009 and March 2010. Patients with AOM and/or acute rhinosinusitis were enrolled into this study without regard for their age, gender, and inpatient or outpatient status, and for whether they were currently undergoing antimicrobial treatment or had undergone such treatment within the past 4 weeks. The diagnostic criteria for AOM were an acute onset of symptoms including ear pain and fever and, for children, crying combined with abnormal tympanic membrane findings such as redness, bulging, and obliteration of landmarks. MEF specimens were collected by ATOMS^®^ tap middle ear aspirator (LUMENIS Co., Ltd., Tokyo, Japan) or by sterile swabs after myringotomy under local anesthesia. The diagnostic criteria for acute rhinosinusitis were an acute onset of symptoms including nasal discharge, headache/irritability, and moist cough combined with postnasal discharge. NPS specimens were also obtained by ATOMS^®^ tap or sterile swabs. Immediately after testing via P6-specific ELISA and plating the specimens for cultures, the samples were stored at −80°C until real-time PCR was performed.

This study was approved by the Institutional Review Board of the Ethical Committee of the Nishinomiya Kyoritsu Neurosurgical Hospital and the Tohoku Rosai Hospital. Before collecting samples, informed consents were obtained by written form from the patients or, for pediatric patients, from their parents or guardians.

### Bacterial cultures

For each bacterial culture, approximately 10 µl of sample was collected by small cotton swabs. The samples were plated on blood and chocolate agar plates and incubated for 24 to 48 h at 37°C under a 5% CO_2_ environment. Bacteria were identified using standard laboratory procedures. *H. influenzae* was identified by growth on chocolate agar, colonial morphology, Gram stain characteristics, and a growth requirement for X and V factors. *S. pneumoniae* was identified by alpha-hemolysis and colonial morphology on 5% sheep blood agar, Gram stain characteristics, optochin sensitivity, and bile solubility. *M. catarrhalis* was identified by colonial morphology, Gram stain characteristics, and the biochemical reaction with butyrate esterase. The two nontypeable *H. influenzae* strains ATCC8149 and ATCC9333 (American Type Culture Collection, Manassas, VA) were used as the control.

### Purification of outer membrane protein P6

The OMP P6 was prepared from *H. influenzae* by a modification that was previously reported elsewhere [16], [17]. Briefly, the outer membrane complex, which consists predominantly of OMPs and lipopolisaccharides, was prepared first. The relative insolubility of P6 in 1% sodium dodecyl sulfate (SDS), 0.1 M Tris, 0.5 M NaCl, 0.1% 2-mercaptoethanol, and a pH of 8.0 (buffer B) was used to separate it from lipooligosaccharides and other OMPs.

Cells of the nontypeable *H. influenzae* ATCC9333 strain cultured on chocolate agar plates overnight at 37°C were harvested and washed with phosphate buffered saline (PBS) several times. The resulting pellet was suspended in buffer B and incubated at 37°C for 30 min with brief sonication to suspend material in buffer B. After having been centrifuged at 21000 g for 30 min at room temperature, the resulting pellet was suspended in buffer B with ribonuclease A (10 µg/ml), incubated at 37°C for 30 min with occasional swirling, and centrifuged at 21000 g for 30 min at room temperature. The pellet was resuspended in borate buffer (0.01 M Tris-HCl, pH 7.4) and incubated at 65°C for 30 min. The insoluble material was removed by centrifugation at 100000 g for 60 min at 30°C. The supernatant was concentrated with centrifugal ultrafiltration using Amicon Ultra-15: MW 3000 cut (EMD Millipore Co., Billerica, MA). The SDS was removed with a surfactant-removing resin. The concentration of P6 in the supernatant was determined by the Bio-Rad Protein Assay (Bio-Rad Laboratories, Hercules, CA). The purification of P6 was confirmed by sodium dodecyl sulfate-polyacrylamide gel electrophoresis (SDS-PAGE) staining with coomassie brilliant blue as a single band around M.W. 16600 dalton, as previously described.

### Preparation of anti-P6–specific polyclonal IgG antibody

Rabbits were subcutaneously immunized with the 100 µg of purified P6 in 0.5 ml of sterile saline emulsified with 0.5 ml of Freund's complete adjuvant 5 times every 2 weeks. Before the 3^rd^ to 5^th^ immunizations, the induction of anti-P6–specific polyclonal IgG antibody was confirmed by usual ELISA. If the levels of anti P6-specific IgG antibody did not reach the plateau, one immunization (the 6^th^ immunization) was boosted.

Two weeks after the final immunization (the 5^th^ or 6^th^ immunization), the immunized rabbits were euthanized and blood for sera was collected. The P6-immunized sera were then first purified with anion exchange using 50% ammonium sulfate precipitation and DEAE Sephadex A-50 (Sigma-Aldrich Co., St. Louis, MO) and were then further purified with Sephadex S300 (Sigma-Aldrich Co.) gel filtration chromatography. The resulting IgG fraction of P6-immunized sera was used as anti-P6–specific polyclonal IgG antibody for the P6-ELISA.

### Procedures for P6-ELISA

A 96-well microtiter plate were coated with 5 µg/ml of purified anti-P6–specific polyclonal IgG antibody overnight at 4°C and blocked with PBS containing bovine serum albumin. The plate was then decanted and supplied with buffers appropriate for the manufacture's P6-ELISA kit for detecting P6 antigen in MEFs and NPSs specimens. The P6 antigen was extracted from specimens by treating them with 1.0 ml of the extraction reagent consisting of phosphate buffer containing detergents and bovine serum albumin.

The P6-ELISA was performed as follows: After rinsing with wash buffer (PBS with detergent), the plates were incubated with 20 µl of the reaction solution (diluted detergent solution) and 100 µl of extracts of specimens or controls at 20°C to 30°C for 60 min with shaking. After washing 6 times with wash buffer, the plates were then incubated with 100 µl of the biotin-conjugated anti-P6 polyclonal IgG antibody at 20°C to 30°C for 60 min with shaking. After washing 6 times with wash buffer, the plates were further incubated with 100 µl of horseradish peroxidase-conjugated streptavidin at 20°C to 30°C for 30 min with shaking. The color was developed with 100 µl of substrate solution containing 3, 3′, 5, 5′-tetramethylbenzidine for 15 min at room temperature after washing 6 times with wash buffer, and the color development was then stopped with diluted sulfuric acid solution. The optical density was measured by a spectrophotometer at 450 nm. The results of P6-ELISA were designated using the following formula: the sample's OD_450_ divided by the mean OD_450_ of the negative control +1/25^th^ of the mean OD_450_ of the positive control based on the result of ROC analysis (data not shown). The P6-ELISA was judged positive when the sample's P6-ELISA value was above 1.0.

The detection limit of the P6-ELISA for purified P6 was 3.13 pg/ml, which was equivalent to 5.2×10^4^ CFU/ml of *H. influenzae* ATCC9333 strains. Cross-reactivity was observed for *H. haemolyticus* at 1.0×10^4^ CFU/ml and for *H. parainfluenzae* at 1.0×10^6^ CFU/ml. No cross-reactivity was found for 53 potential cross-reactants including 35 microbes and 19 viruses (manufacture's data).

### Real-time PCR for quantifying the *ompP1* gene

The relative amount of *H. influenzae* DNA genome was quantified by real-time PCR using primers and the TaqMan probe established for the region of the *ompP1* gene of *H. influenzae*. Briefly, total genomic DNA was extracted by the QIAamp DNA Mini Kit (QIAGEN, Valencia, CA). Real-time PCR proceeded on thermal cycler ABI7700 or ABI7900 (Applied Biosystems, Foster City, CA). The nucleotide sequences of the primers and of the carboxyfluorescein-labeled probe were as follows: forward primer: 5′-ccttacgtgcRggtatKgct-3′, reverse primer: 5′-gtgaacttttttgccttttaagtaagc-3′, and carboxyfluorescein-labeled probe: 5′-(FAM)-agtgctgcaattccagataccgatcgc-(TAMRA)-3′. RNase-free water and DNA extracted from the *H. influenzae* ATCC9333 strain were used for negative and positive controls, respectively. After an initial denaturation at 95°C for 15 min, the PCR reaction was followed by 50 cycles of amplification at 94°C for 15 sec and at 60°C for 1 min. The number of copies of the *ompP1* gene was calculated. If no increase in the fluorescent signal was observed after 50 cycles, the sample was regarded as negative. Positive and negative controls were included at every extraction and at every PCR run. The limit of the assay is 40 copies of DNA.

### Statistics

Statistical analysis was done by Prism 5 (GraphPad Software, Inc., La Jolla, CA). The influenzae DNA densities were compared by the Mann-Whitney U test. A *p*-value of ≤0.05 was considered statistically significant. Ninety-five percent confidential intervals (CIs) were calculated.

## Results

### Populations

The population for this study consisted of 257 patients with AOM (244 with simple AOM and 13 with intractable OM) and 265 patients with acute rhinosinusitis (259 with acute rhinosinusitis and 6 with acute exacerbation of chronic sinusitis). Of the patients in the study, 197 had both AOM and acute rhinosinusitis simultaneously.

Patients with AOM ranged in age from 0 to 56 years old, had a median age of 1 year, and included 112 females and 145 males. The AOM patients included 250 children (0 to 14 years old) and 7 adults (18 to 56 years old). Patients with acute rhinosinusitis ranged in age from 0 to 75 years old, had a median age of 1 year, and included 122 females and 143 males. The rhinosinusitis patients included 247 children (0 to 14 years old) and 18 adults (18 to 75 years old). Because of the small number of adult samples, we analyzed both MEF and NPS samples without regarding the ages of the patients. Finally, we obtained 257 MEFs including 13 otorrhea samples from patients with AOM and 265 NPSs from patients with acute rhinosinusitis.

### Bacterial cultures

When the samples were tested via conventional bacterial cultures, *H. influenzae* was identified in 90 (35.0%) out of 257 MEFs. Out of these 90 strain samples, 80 strains were a single pathogen and 10 strains were a combination of pathogens. Ten MEFs contained *H. influenzae* combined with either or both *S. pnuemoniae* and *M. catarrhalis*. (Six MEFs contained *S. pnuemoniae*, 1 MEF contained *M. catarrhalis*, and 1 MEF contained both *S. pneumonia* and *M. catarrhalis*). *S. pneumoniae* and *M. catarrhalis* were identified in 59 (23.0%) MEFs and 9 (3.5%) MEFs, respectively. Ninety-six (37.4%) of the MEFs did not contain pathogenic bacteria.

Out of 265 NPSs, *H. influenzae* was identified in 156 (58.9%) samples. Thirty-one NPSs contained *H. influenzae* as a single pathogen, and 125 MEFs contained *H. influenzae* combined with either or both *S. pnuemoniae* and *M. catarrhalis.* (Forty-one NPSs contained *S. pneumoniae*, 28 NPSs contained *M. catarrhalis,* and 55 contained both pathogens.) *S. pneumoniae* and *M. catarrhalis* were identified in 126 (47.5%) NPSs and 97 (36.6%) NPSs, respectively. Nineteen samples (7.2%) showed no growth of pathogenic bacteria.

### Quantification of the *ompP1* gene in middle ear fluids and nasopharyngeal secretions

**Figure 1:**
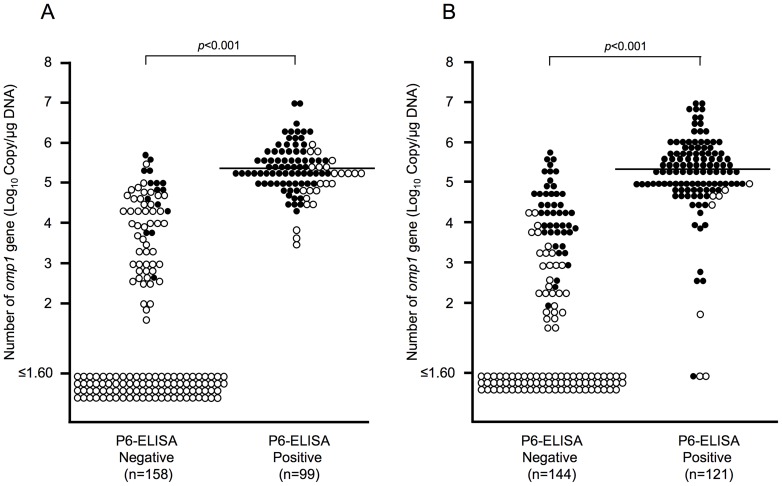
Distribution of the number of copies of the *H.* *influenzae ompP1* gene in MEFs and NPSs based on the results of P6-ELISA and conventional culture. Vertical axis: the number of copies of the *ompP1* gene calculated from real-time PCR. Open circle: *H. influenzae* culture negative, closed circle: *H. influenzae* culture positive. The number of copies of the *H. influenzae ompP1* gene in ODK-0902-positive arid culture-positive populations differed significantly between ODK-0902-positive and -negative populations (*p*<0.001).

We used real-time PCR to detect and quantify the relative amount of *H. influenzae* in the MEFs and NPSs. The number of *ompP1* gene copies was significantly higher in MEFs positive for the P6-ELISA than in the MEFs negative for P6-ELISA (2.0×10^5^ vs. <40 copies/µg DNA, *p*<0.01) (Fig. 1A). The number of *ompP1* gene copies in MEFs was also significantly higher in culture-positive specimens than in culture-negative specimens (*p*<0.001). The 64 (40.5%) out of 158 MEF samples that were negative for P6-ELISA presented the *ompP1* gene, and 15 (9.5%) of them were culture positive. In contrast, all of the 99 MEFs positive for P6-ELISA contained the *ompP1* gene, and 24 (24.2%) of them were negative by culture.

The number of *ompP1* gene copies in NPSs was also significantly higher in samples positive for P6-ELISA than in samples negative for the test (2.0×10^5^ vs. <40 copies/µg DNA, *p*<0.01) (Fig. 1B). The number of *ompP1* gene copies in the NPSs was significantly higher in culture-positive specimens than in culture-negative specimens (*p*<0.001). Among the 121 NPSs positive by P6-ELISA, 118 samples (97.5%) were positive for the *ompP1* gene. The remaining three samples were P6-ELISA positive but were negative for the *ompP1* gene, and one of them was positive by culture. On the other hand, among the 144 NPSs negative by P6-ELISA, 73 (50.7%) samples had the *ompP1* gene and 45 (31.3%) of them were positive by culture.

### Sensitivity and specificity of P6-ELISA for MEFs and NPSs

OMP P6 was detected in 99 MEFs (38.5%, 95% CI 0.33–0.46) and 121 NPSs (45.7%, 95% CI 0.40–0.52). When bacterial culture was used as the gold standard, the sensitivity and specificity of P6-ELISA for identifying P6 in MEFs were 83.3% (95% CI 0.76–0.91) and 85.6% (95% CI 0.80–0.91), respectively. The positive predicting value (PPV) and negative predictive value (NPV) of the test for MEFs were 75.8% (95% CI 0.67–0.84) and 90.5% (95% CI 0.86–0.95), respectively (Table 1, Appendix S1). The sensitivity and specificity of the P6-ELISA for identifying P6 in NPSs were 71.5% (95% CI 0.65–0.79) and 92.5% (95% CI 0.88–0.98), respectively. The PPV and NPV of the test for NPSs were 93.4% (95% CI 0.89–0.98) and 68.8% (95% CI 0.61–0.76), respectively (Table 1, Appendix S1).

**Table 1:** Sensitivity, specificity, positive predicting value, and negative predicting values of P6-ELISA to determine the presence of *H. influenzae* in MEFs and NPS.

Category	For NPSs			For MEFs		
	Total	With prior antimicrobial treatment	Without antimicrobial treatment	Total	With prior antimicrobial treatment	Without antimicrobial treatment
**Sensitivity**	71.5% (113/158)	61.2% (30/49)	76.1% (83/109)	83.3% (75/90)	66.7% (12/18)	87.5% (63/72)
**Specificity**	92.5% (99/107)	95.2% (20/21)	91.9% (79/86)	85.6% (143/167)	79.3% (46/58)	89.0% (97/109)
**PPV**	93.4% (113/121)	96.8% (30/31)	92.2% (83/90)	75.8% (75/99)	50.0% (12/24)	84.0% (63/75)
**NPV**	68.8% (99/144)	51.3% (20/39)	75.2% (79/105)	90.5% (143/158)	88.5% (46/52)	91.5% (97/106)

The numbers in a parentheses show the number of samples.

Furthermore, the performance of P6-ELISA based on the presence or absence of previous antibiotic treatment was evaluated. The sensitivity, specificity, PPV, and NPV of P6-ELISA for MEFs from patients with prior antimicrobial treatment were 66.7% (95% CI 0.45–0.88), 79.3% (95% CI 0.69–0.90), 50.0% (95% CI 0.30–0.70), and 88.5% (95% CI 0.80–0.97), respectively. On the other hand, the results for MEFs from patients without prior antimicrobial treatment were 87.5% (95% CI 0.80–0.95), 89.0% (95% CI 0.83–0.95), 84.0% (95% CI 0.76–0.92), and 91.5% (95% CI 0.86–0.97), respectively (Table 1, Appendix S1). For NPSs, the sensitivity, specificity, PPV, and NPV from patients with prior antimicrobial treatment were 61.2% (95% CI 0.48–0.75), 95.2% (95% CI 0.86–1.04), 96.8% (95% CI 0.91–1.03), and 51.3% (95% CI 0.36–0.67), respectively. The results for NPSs from patients without prior antimicrobial treatment were 76.1% (95% CI 0.68–0.84), 91.9% (95% CI 0.86–0.98), 92.2% (95% CI 0.87–0.98), and 75.2% (95% CI 0.67–0.83), respectively (Table 1, Appendix S1).

### Evaluating the ability of P6-ELISA of NPSs to positively and negatively predict *H. influenzae* in the middle ear

For 203 cases of AOM, we compared the abilities of P6–ELISA and conventional bacterial cultures of NPSs to accurately make bacteriologic assessments of the middle ear pathogen. The PPV and NPV of using conventional nasopharyngeal bacterial cultures to detect the presence of *H. influenzae* in MEFs were 49.6% (95% CI: 41.1%–58.1%) and 92.9% (95% CI: 86.8%–98.9%), respectively. In contrast, the PPV and NPV of using the nasopharyngeal P6-ELISA to detect the presence of *H. influenzae* in MEFs were 57.3% (95% CI: 47.4%–67.2%) and 85.0% (95% CI: 78.3%–91.8%), respectively (Table 2).

**Table 2:** Positive and negative predicting values resulting from evaluating NPSs to determine the presence of *H. influenzae* in MEFs.

Category	Nasopharyngeal secretion	
	Culture	ODK-0902
**Sensitivity**	93.0% (66/71)	77.5% (55/71)
**Specificity**	49.2% (65/132)	68.9% (91/132)
**Agreement rate**	64.5% (131/203)	71.9% (146/203)
**Positive predictive value**	49.6% (66/133)	57.3% (55/96)
**Negative predictive value**	92.9% (65/70)	85.0% (91/107)

## Discussion

While the rapid antigen detection test is a successful diagnostic tool for pneumococci, that test is not yet useful for nontypeable *H. influenzae* [18]–[21]. To our knowledge, this is the first study that has evaluated the applicability of P6-ELISA for diagnosing AOM and acute rhinosinusitis in children and adults caused by nontypeable *H. influenzae*.

When compared with results from bacterial cultures, P6-ELISA yields a favorable sensitivity and specificity of 83.3% and 85.6% for MEFs and 71.5% and 92.5% for NPSs, respectively. In comparison with the results for MEFs, P6-ELISA showed a lower specificity and sensitivity for NPSs. Because *H. influenzae* can be indigenous to the human nasopharynx, NPSs might frequently be positive by culture [22]. In this study, we conducted a further quantitative evaluation of *H. influenzae* using P6-ELISA. P6-ELISA is a qualitative test that has adequate volume proportionality because it is able to detect a significant difference between the number of copies of the *ompP1* gene in positive and negative samples. Most of the specimens that were positive via P6-ELISA were also positive via bacterial cultures and had more copies of the *ompP1* gene according to real-time PCR. These findings suggested that P6-ELISA will not detect and thus not be hindered by the small amounts of *H. influenzae* in NPSs, which translates into a low false-positive rate for P6-ELISA in the presence of indigenous *H. influenzae*.

In this study, false-positive results were obtained for P6-ELISA in 23.2% (23/99) of MEFs and in 5.8% (7/121) of NPSs. All MEFs samples with false-positive results via P6-ELISA contained the *ompP1* gene even though the results via bacterial culture showed negative results. These finding suggested the presence of *H. influenzae* in MEFs. However, P6-ELISA also found that three NPSs were positive for *H.* influenzae even though they did not contain the *ompP1* gene. Significantly, P6-ELISA reacts with *H. haemolyticus* and *H. parainfluenzae*, both of which are phylogenetically closely related to *H. influenzae* and frequently reside as a commensal organism in the nasopharynx [23]–[25]. The recent discovery of nonhemolytic *H. haemolyticus* further increases the difficulty of distinguishing NTHi from *H. haemolyticus* [26], [27]. Although multilocus sequence typing (MLST) provides the most accurate identification of true NTHi, that technique is not practical for routine clinical use because of its high expense and labor requirements [28], [29]. Several studies have focused on the identification of a single gene target for accurate diagnosis of NTHi from *H. haemolyticus* [27]–[33]. Some of these single gene targets are the lipo-oligosaccharide gene *lgtC*, the IgA protease gene *iga*, the fuculose kinase gene *fucK*, the pilus gene *pilA*, the 16S rRNA gene, and protein D gene *hpd*.

However, it is not yet possible to absolutely distinguish NTHi from *H. hemolyticus*. In a previous very detailed examination, 27% of pharyngeal isolates from obstructive pulmonary disease and 12% of naspharyngeal isolates from otitis-prone children phenotypically defined as NTHi were instead identified as *H. haemolyticus* by 16S rRNA gene PCR [27], [34]. In contrast to the relatively high prevalence of *H. haemlyticus* in the nasopharynx, other studies demonstrated that none out of 250 middle ear isolates phenotypecally defined as NTHi were actually *H. haemolyticus* or nonhaemolytic variant strains [23], [27], [30]. In this study, the NPVs of P6-ELISA for MEFs were higher than those for NPSs. However, the use of P6-ELISA to accurately detect the presence of NTHi is controversial [34]. Although the prevalence of these non-pathogenic *Haemophilus* species is low, further study is necessary to resolve these problems.

In contrast, false negatives were found in 8.9% (14/158) of MEFs and 30.5% (44/144) of NPSs. However, all samples that P6-ELISA found to be false negative contained the *ompP1* gene. Degeneration of P6 may decrease the reactivity of P6-ELISA. Furthermore, the considerable heterogeneity of P6 expression among *H. influenzae* clinical isolates could cause a significant variation of false negatives [35], [36].

Finally, we examined NPSs to evaluate their use in determining the PPV and NPV of middle ear pathogens. While the prediction of the causative agent for moderate and more severe otitis media is possible through the use of myringotomies to collect MEFs from the resulting drainage, MEFs are sometimes not available: Middle ear fluid is often difficult to collect because acute otitis media frequently strikes in children for whom general anesthesia is preferred prior to myringotomies. Acute otitis media is thought to occur when causative agents that accumulated in the nasopharynx invade the middle ear via the Eustachian tubes. Therefore, the ability to identify the bacteria in the nasopharynx increases the rate at which the type of bacteria in middle ear fluid can accurately be predicted [37]–[39]. However, when used to analyze nasopharyngeal secretions, P6-ELISA showed predictive rates for bacteria in middle ear comparable to the predictive rates from bacterial culture.

In conclusion, early diagnosis and appropriate antimicrobial treatment comprise the basic principles of treating infectious disease. However, the current P6-ELISA test has limitations as a method for the exact detection of NTHi and is thus a weak method for distinguishing NTHi from the two commensals. The merits of P6-ELISA are somewhat weak for diagnosis. Consequently, its use as a diagnostic tool could result in misleading conclusions concerning the specific cause of patient symptoms if it is used in clinical settings. Thus, we pay caution of the misleading if the P6-ELISA is used for diagnosis. In contrast, an advantage of ELISA is the stability of its ability to detect target antigens in samples with or without prior antimicrobial treatment while the ability of standard culture techniques was hampered by previous antimicrobial treatments. Thus, there might be value in using ELISA to detect NTHi if the appropriate single target antigen were applied for distinguishing NTHi from the two commensals.

## Supporting Information

Appendix S1 **Sensitivity and specificity of P6-ELISA for MEFs with and without prior antimicrobial treatments.**
(DOC)
