# Supplementary material for: An Application of Outer Membrane Protein P6-Specific Enzyme-Linked Immunosorbent Assay for Detection of Haemophilus influenzae in Middle Ear Fluids and Nasopharyngeal Secretions
Source: PLoS One. 2013 Aug 28;8(8):e71774. doi: 10.1371/journal.pone.0071774 (PMC3756020; doi:10.1371/journal.pone.0071774)
Supplement: Appendix S1 — Sensitivity and specificity of P6-ELISA for MEFs with and without prior antimicrobial treatments. (DOC) [file pone.0071774.s001.doc]

Appendix 1. Sensitivity and specificity of P6-ELISA for MEFs with and without prior antimicrobial treatments.

| **P6-ELISA** | **Culture from NPSs** | | | | | | **Culture from MEFs** | | | | | |
| --- | --- | --- | --- | --- | --- | --- | --- | --- | --- | --- | --- | --- |
|  | With prior antimicrobial treatment | | | Without prior antimicrobial treatment | | | With prior antimicrobial treatment | | | Without prior antimicrobial treatment | | |
|  | Positive | Negative | total | Positive | Negative | total | Positive | Negative | total | Positive | Negative | total |
| Positive | 30 | 1 | 31 | 83 | 7 | 90 | 12 | 12 | 24 | 63 | 12 | 75 |
| Negative | 19 | 20 | 39 | 26 | 79 | 105 | 6 | 46 | 52 | 9 | 97 | 106 |
| Total | 49 | 21 | 70 | 109 | 86 | 195 | 18 | 58 | 76 | 72 | 109 | 181 |
